# Evaluating Tailored Learning Experiences in Emergency Residency Training Through a Comparative Analysis of Mobile-Based Programs Versus Paper- and Web-Based Approaches: Feasibility Cross-Sectional Questionnaire Study

**DOI:** 10.2196/57216

**Published:** 2025-07-24

**Authors:** Hsin-Ling Chen, Chen-Wei Lee, Chia-Wen Chang, Yi-Ching Chiu, Tzu-Yao Hung

**Affiliations:** 1 Department of Emergency Medicine, Zhong-Xing branch, Taipei City Hospital, 145 Zhengzhou Road, Taipei, 10341, Taiwan, 886 025522915; 2 Department of Emergency, Dalin Tzu Chi Hospital, Buddhist Tzu Chi Medical Foundation, Chiayi County, Taiwan; 3 School of Medicine, Tzu Chi University, Hualien County, Taiwan; 4 Department of Surgery, Zhong-Xing branch, Taipei City Hospital, Taipei, Taiwan; 5 Faculty of Medicine, National Yang Ming Chiao Tung University, Taipei, Taiwan

**Keywords:** app, mobile, web-based, competency-based medical education, residency training program

## Abstract

**Background:**

In the rapidly changing realm of medical education, Competency-Based Medical Education is emerging as a crucial framework to ensure residents acquire essential competencies efficiently. The advent of mobile-based platforms is seen as a pivotal shift from traditional educational methods, offering more dynamic and accessible learning options. This research aims to evaluate the effectiveness of mobile-based apps in emergency residency programs compared with the traditional paper- and web-based formats. Specifically, it focuses on analyzing their roles in facilitating immediate feedback, tracking educational progress, and personalizing the learning journey to meet the unique needs of each resident.

**Objective:**

This study aimed to compare mobile-based emergency residency training programs with paper- and web-based (programs regarding competency-based medical education core elements.

**Methods:**

A cross-sectional web-based survey (Nov 2022-Jan 2023) across 23 Taiwanese emergency residency sites used stratified random sampling, yielding 74 valid responses (49 educators, 16 residents, and 9 Residency Review Committee hosts). Data were analyzed using Mann-Whitney *U* test, chi-squared tests, and *t* tests.

**Results:**

MB programs (n=14) had fewer missed assessments (*P*=.02) and greater ease in identifying performance trends (*P*<.001) and required clinical scenarios (*P*<.001) compared with paper- and web-based programs (n=60). In addition, mobile-based programs enabled real-time visualization of performance trends and completion rates, facilitating individualized training (*P*<.001).

**Conclusions:**

In our nationwide pilot study, we observed that the mobile-based interface significantly enhances emergency residency training. It accomplishes this by providing rapid, customized updates, thereby increasing satisfaction and autonomous motivation among participants. This method is markedly different from traditional paper- or web-based approaches, which tend to be slower and less responsive. This difference is particularly evident in settings with limited resources. The mobile-based interface is a crucial tool in modernizing training, as it improves efficiency, boosts engagement, and facilitates collaboration. It plays an essential role in advancing Competency-Based Medical Education, especially concerning tailored learning experiences.

## Introduction

The competency-based medical education (CBME) model has been a global trend in residency training for over a decade [1-4], with timely, personalized, and meaningful coaching feedback as one of its core elements [4]. At the same time, advances in mobile technology are reshaping both medical education and clinical practice [5-11]. Mobile learning has emerged as a cost-effective, accessible approach that supports context-driven, real-time learning and continuous feedback—despite challenges like technical limitations and potential distractions [5]. A national survey indicates that tablet use, predominantly iPads, is on the rise, with strong support for further integration to enhance clinical efficiency, particularly among younger clinicians [8]. In addition, guidelines for mobile technologies in workplace-based assessments show that these devices can streamline real-time data capture, reduce administrative burdens, and facilitate competency-based decision-making through intuitive interfaces, robust security, and comprehensive training [12].

A residency training program is designed to help residents manage patients effectively across diverse scenarios while continuously improving their skills. The goal is to foster intrinsic motivation and enhance performance by creating an environment that supports autonomy, competence, and relatedness [13].

Repeated, multisource evaluations provide a more reliable trainee assessment than one-off reviews [14,15]. Oudkerk Pool et al [14] found that multiple evaluators help mitigate biases by iteratively acquiring, organizing, and integrating evidence. As CBME emphasizes longitudinal performance tracking, effective data management is crucial. Leveraging big data enables objective, evidence-based promotion decisions, reducing reliance on subjective faculty recall [16].

However, performance evaluation demands extensive documentation, data collection, and analysis, which can be time-consuming and resource-intensive, potentially affecting the quality of feedback. In addition, interpreting performance trends is complex and must generate meaningful insights for program committees to tailor and individualize training. For resource-limited programs, the infrastructure required to integrate evaluation interfaces and visualize performance trends across trainers, trainees, and program committees poses a significant challenge [5,12,16].

**Figure 1.**
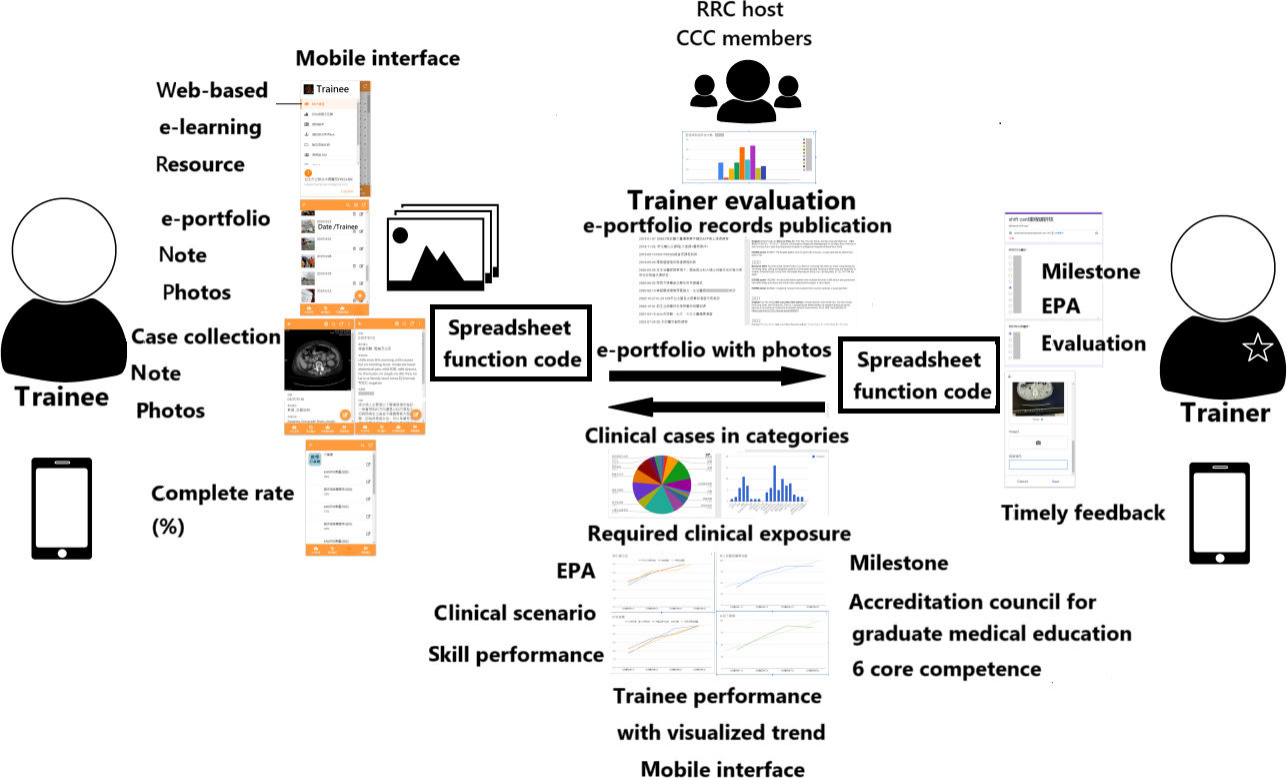
The illustration of the mobile-based interface is shared among trainers, trainees, the Residency Review Committee host, and Clinical Competency Committee members. CCC: Clinical Competency Committee; EPA: entrustable professional activities; RRC: Residency Review Committee.

Although mobile apps for medical learning have advanced significantly [5-7,9,17,18], assessment in training programs has largely relied on paper- or web-based systems [19]. However, computers and laptops can be cumbersome and require stable internet access, which is not always available. In contrast, mobile interfaces on smartphones and tablets offer a more accessible and user-friendly alternative without compromising content quality (Figure 1) [5-9,12]. These platforms facilitate frequent performance monitoring and review, promoting continuous improvement in alignment with evolving educational and professional needs [12].

**Figure 2.**
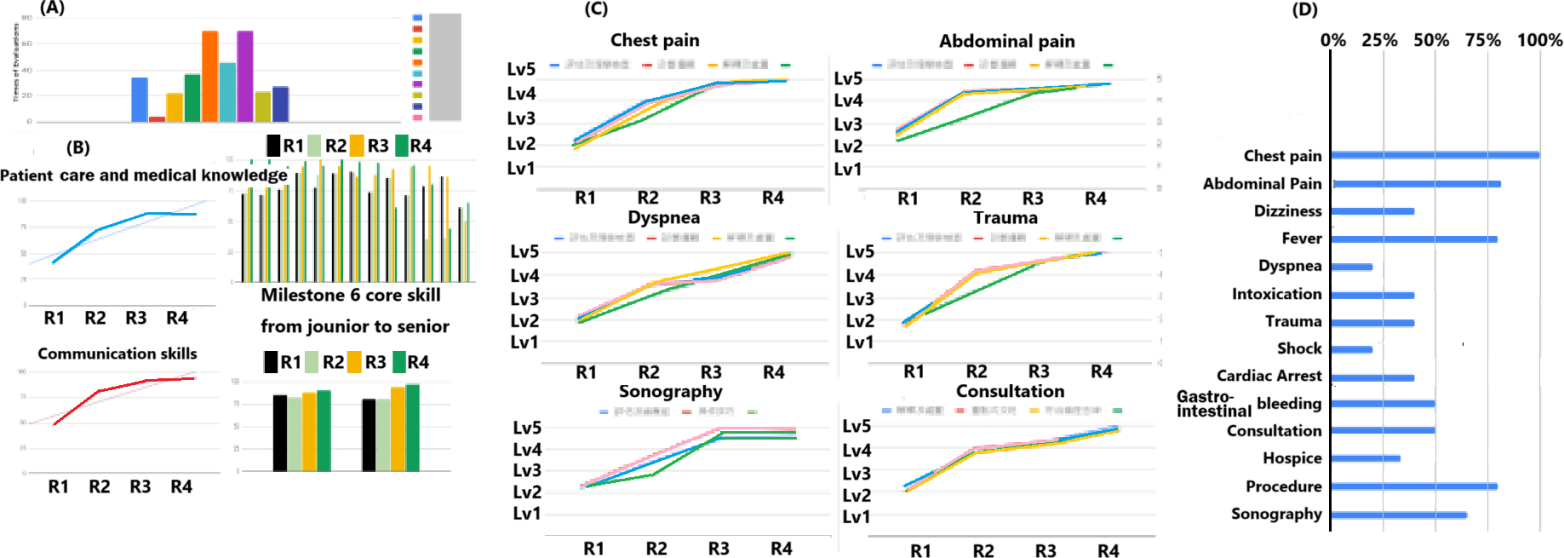
(A) The assessment distribution by the trainer is based on one training site with a mobile-based assessment system. The trainee's milestones are assessed weekly, and these data can be accumulated and transformed into visualized trends quarterly and annually to track progress. (B) Entrustable professional activities, with scenarios characterized by varying levels of emergency, can be accumulated and transformed into visualized trends quarterly and annually to gauge progress. (C) The required assessments of entrustable professional activities can be converted into a completion rate to aid trainers in more comprehensive assessments of trainees. EPA: entrustable professional activity.

Ideally, residents and faculty can exchange real-time feedback, allowing both groups to track performance trends and refine learning and coaching strategies. Rather than relying on one-time scores, the Residency Review Committee (RRC) and Clinical Competency Committee (CCC) can assess performance through visual trend analyses (Figure 2), enabling personalized program adjustments and targeted interventions for underperformance [12]. This framework consolidates evaluations from multiple sources, transforming data into motivational insights for trainees and actionable trends for program directors. In addition, visualizing progressive percentages helps trainees understand their clinical diversity requirements and ensures they receive adequate competency evaluations throughout emergency residency training.

Workplace-based assessments are a cornerstone of CBME, particularly in emergency residency training, where timely feedback and performance tracking are essential [12]. While paper-based and some web-based platforms (eg, Google Forms and SurveyCake) facilitate structured evaluations, they often present challenges in real-time data integration, accessibility, and administrative burden for trainers and trainees.

Despite advancements in medical education technology, our preliminary data from Taiwan suggest that mobile interface assessments remain significantly underused compared with web-based alternatives, with an approximate ratio of 1:4-1:5. This disparity may reflect institutional technological barriers, faculty adoption challenges, or security and interoperability concerns, all of which hinder the seamless aggregation and visualization of multisource evaluation data. While web-based platforms are widely implemented, the potential of mobile-based platforms to enhance real-time feedback and streamline competency tracking in residency training remains underexplored [16,17].

This pilot study aims to determine whether mobile-based platforms can address these challenges by improving real-time performance monitoring and optimizing feedback mechanisms in emergency residency training. By evaluating user adoption, data integration efficiency, and feedback effectiveness, this study seeks to provide insights into the feasibility and impact of mobile technology in workplace-based medical assessments.

## Methods

### Overview

A cross-sectional, web-based survey was conducted from November 3, 2022, to January 3, 2023, to explore the perspectives of educators and resident physicians on assessment platforms in emergency residency training. As a preliminary investigation, this study aimed to assess the feasibility of mobile-based assessments, refine survey methodology, and identify key themes for future large-scale research.

We calculated the sample size using a *t* test for 2 independent proportions. Given that mobile-based programs constitute approximately one-fourth of RRC programs, we based our estimation on a 1:4 ratio. Assuming a 1-point difference in evaluations regarding training individualization, we determined that a minimum of 11 mobile-based participants and 44 paper- and web-based participants would be needed to achieve 80% power at *α*=.05 [20].

The survey targeted program hosts, trainers, and trainees across emergency residency training sites in Taiwan, inviting them to participate anonymously (Multimedia Appendix 1). A stratified random sampling method was applied, proportionally selecting participants from accredited emergency residency training hospitals as recognized by the National Emergency Medicine Association. From a total of 1068 qualified educators and 313 resident physicians, a 5% sampling rate was used, resulting in invitations sent to 55 educators and 20 resident physicians. In addition, to gather insights on the perspectives of RRC hosts, 10 were invited to participate in the survey [20].

This study evaluates assessment designs used across different emergency residency training sites. Participants from diverse programs were categorized into paper- and web-based or mobile-based evaluation methods for detailed analysis. Paper- and web-based programs were grouped together due to shared limitations, such as delayed feedback, lack of real-time performance tracking, and reliance on periodic evaluations rather than continuous competency monitoring. In contrast, mobile-based programs provided immediate feedback and seamless trainee progress tracking, distinguishing them as a separate category.

The questionnaire was developed using the Delphi method, ensuring expert consensus on its content and structure. It included a background survey capturing details, such as the name and level of the training site, the number of trainers and trainees, and the participant’s role in the training program. The core survey items consisted of Likert-scale questions (5-point scale, ranging from “Strongly Disagree” to “Strongly Agree”) designed to assess perceptions of CBME core elements. These responses were analyzed and compared to evaluate differences in assessment approaches. To ensure the integrity and reliability of the collected data, only fully completed surveys were included in the final analysis. Incomplete responses were excluded to maintain data accuracy. In addition, responses underwent internal consistency checks to detect and exclude any contradictory or inconsistent answers.

The questionnaire was distributed on the web, with participants invited via email to complete it voluntarily and anonymously. No monetary incentives were provided; however, participants were informed that their input would contribute to improving workplace-based assessments in emergency residency training. While hosts offered additional insights, their responses were categorized under trainers for analysis, as their primary role aligned with faculty responsibilities.

### Statistical Analysis

Analyses were conducted using IBM SPSS Statistics (version 23). Dependent variables included assessment satisfaction, assessment and feedback duration, frequency of missed assessments, ability to identify performance trends, ability to review and respond within 24 hours, ability to individualize training programs based on performance results, and ability to track the completion rate of required clinical scenarios and assessments.

Independent variables included the training site level (district, regional, or center hospitals), participants' age, participant role (trainee or trainer), and assessment platform (mobile-based or paper- and web-based).

The Mann-Whitney *U* test was used to compare differences in dependent variables across training sites and participant roles. A linear regression model with repeated measurements at the hospital level was applied to evaluate the ability to individualize training programs based on performance results, adjusting for potential confounders, including the number of trainers and trainees. The chi-squared test was used to analyze differences in assessment platform performance, with statistical significance set at *P*<.05. The chi-square test was used to analyze differences in assessment platform performance, with statistical significance set at *P*<.05.

### Ethical Considerations

This pilot study received ethical approval from the Taipei City Hospital institutional review board (TCHIRB-11110007-E). The study was conducted after being approved by the Taipei City Hospital institutional review board on November 24, 2022. The survey was anonymous. The informed consent was waived by the Taipei City Hospital institutional review board.

## Results

### Demographics

In this study, 62.22% of emergency residency training sites nationwide participated, amounting to 28 out of 45 sites. A total of 74 valid responses were collected from 28 emergency residency training sites across Taiwan, including district, regional, and center hospitals. Among the respondents, 49 trainers participated, reflecting an 89.1% response rate, while 16 resident trainees responded, yielding an 80% response rate. To further explore the perspectives of RRC hosts, 10 were invited to participate, with 9 providing responses. Regarding assessment platform usage, 14 participants reported using mobile-based assessments, whereas the remaining 60 relied on either paper- or web-based platforms. Within this group, 3 participants used paper-based assessments, and 57 used web-based systems.

### Survey Validity Results

The questionnaire demonstrated strong reliability for trainee responses, with a Cronbach α of 0.83, indicating high internal consistency. For trainers, the reliability was moderate, with a Cronbach α of 0.61, which is acceptable for exploratory research. In addition, the content validity index of 0.96 confirmed strong expert agreement on item relevance, supporting the overall validity of the questionnaire.

### Survey Results

The analysis revealed no significant differences between trainers and trainees concerning the distribution of assessment platforms, satisfaction with the evaluation process, the time taken for evaluation and feedback, the likelihood of forgetting mutual assessments, or responses to core CBME-related questions (Table 1). However, when comparing mobile-based assessments with paper- and web-based platforms, mobile interfaces demonstrated several advantages. Respondents using mobile-based platforms reported higher satisfaction with the assessment process, a lower likelihood of missing assessments, and an improved ability to identify performance trends. In addition, they were more likely to review feedback within 24 hours, found it easier to tailor training programs based on performance results, and experienced greater ease in identifying required clinical scenarios and necessary assessments (Table 2).

**Table 1.** Characteristics of internet survey participants from emergency residency training sites. Data are presented as N (%), mean (SD), or median (IQR).

Variables	Total (N=74)	Group		*P* value
(%)	Trainer, (n=58)	Trainee, (n=16)
The level of the training site, n (%)				—^a^
District hospital	3 (4)	2 (4)	1 (6)	
Regional hospital	45 (61)	33 (61)	12 (75)	
Medical center	26 (35)	23 (35)	3 (19)	
Numbers of trainer in the training site				—
Mean (SD)	16.6 (9.9)	18.1 (10.5)	11.1 (3.3)	
Median (IQR)	13 (3-23)	15 (5-25)	10 (9-11)	
Numbers of trainee in the training site				—
Mean (SD)	7.5 (5.2)	8.2 (5.3)	4.9 (3.7)	
Median (IQR)	5 (1-9)	7 (3-11)	4 (2-6)
Group, n (%)				.49
Paper and web (Google, Survey Cake etc.)	60 (81)	48 (83)	12 (75)	
Mobile-based	14 (19)	10 (17)	4 (25)	
Assessment interface, n (%)				.46
Mobile-based	14 (19)	10 (17)	4 (25)	
Paper-based	3 (4)	1 (2)	2 (13)	
Web-based	57 (77)	47 (81)	10 (62)	
Degree of satisfaction, n (%)				.06
Very satisfied	12 (16)	8 (14)	4 (25)	
Satisfied	27 (36)	22 (38)	4 (25)	
Neutral	26 (35)	22 (38)	5 (31)	
Dissatisfied	7 (9)	6 (10)	1 (6)	
Very dissatisfied	2 (3)	0 (0)	2 (13)	
The duration of assessment process in each shift (min)				.08
Mean (SD)	16 (10)	16 (11)	13 (9)	
Median (IQR)	15 (5-15)	15 (5-15)	10 (2.5-22.5)
The duration of feedback in each shift (min) (orally or in text)				.77
Mean (SD)	19 (16)	20 (17)	17 (13)	
Median (IQR)	15 (5-15)	15 (5-15)	15 (5-15)
Likelihood of forgetting to complete the assessments				.11
Mean (SD)	3.1 (1.3)	3.2 (1.3)	2.6 (1.3)	
Median (IQR)	3 (1-5)	3 (1-5)	3 (1-5)
Present Method of the performance result, n (%)				
Not seen	4 (5)	2 (3)	2 (13)	.2
With number (0-100)	17 (23)	14 (24)	3 (19)	.75
With level (1-5)	53 (72)	41 (70)	12 (75)	>.99
With number and visualized trend	31 (42)	23 (40)	8 (50)	.57
With number, visualized trend, and completion rate	36 (49)	28 (48)	8 (50)	>.99
Can you identify whether the performance trend is improved or worsened from the assessment result?				.95
Mean (SD)	3.1 (1.3)	3.1 (1.2)	3.1 (1.5)	
Median (IQR)	3 (1-5)	3 (1-5)	3 (1-5)
Can you review and respond to the feedback within 24 h?				.35
Mean (SD)	2.7 (1.3)	2.6 (1.1)	3.0 (1.6)	
Median (IQR)	3 (1-5)	3 (1-5)	3 (1-5)
Are you able to individualize the training program based on performance results?				.4
Mean (SD)	2.7 (1.1)	2.7 (1.1)	2.5 (1.4)	
Median (IQR)	2 (1-3)	2 (1-3)	3 (1-5)
Can you identify the required clinical scenarios and the assessments needed for each trainee?				.24
Mean (SD)	2.6 (1.3)	2.6 (1.2)	2.4 (1.5)	
Median (IQR)	2 (1-3)	2 (1-3)	2 (1-3)

a Not applicable.

**Table 2.** The comparison between mobile-based and paper or web-based programs.

Variables	Total	Group		*P* value
	(n=74)	Paper or Web (n=60)	Mobile (n=14)	
The level of the training site, n (%)				—^a^
District hospital	3 (4)	1 (2)	0 (0)	
Regional hospital	45 (61)	32 (53)	14 (100)	
Medical center	26 (35)	26 (43)	0 (0)	
Numbers of trainer in the training site				<.001
Mean (SD)	16.6 (9.9)	18.1 (10.4)	10.0 (0.0)	
Median (IQR)	13 (3-23)	15 (6-24)	10 (10-10)	
Numbers of trainee in the training site				.04
Mean (SD)	7.5 (5.2)	8.1 (5.6)	4.9 (0.4)	
Median (IQR)	5 (1-9)	8 (1-15)	5 (5-5)	
Participant age (year-old)				.65
Mean (SD)	42.1 (8.2)	41.3 (8.4)	42.0 (7.9)	
Median (IQR)	43 (31-55)	42 (31.5-52.5)	45 (35.5-54.5)	
Participant roles, n (%)				.53
Junior resident (R1/R2)	7 (9)	5 (8)	2 (14)	
Senior resident (R3/R4)	9 (12)	7 (12)	2 (14)	
RRC^b^ host	9 (12)	8 (13)	1 (7)	
Chief of the department	9 (12)	9 (15)	0 (0)	
Clinical instructor	40 (54)	31 (52)	9 (64)	
Assessment interface, n (%)				—
Mobile-based	14 (19)	0 (0)	14 (100)	
Paper-based	3 (4)	3 (5)	0 (0)	
Web-based	57 (77)	57 (95)	0 (0)	
Degree of satisfaction, n (%)				<.001
Very satisfied	12 (16)	3 (5)	9 (64)	
Satisfied	27 (36)	22 (37)	5 (36)	
Neutral	26 (35)	26 (43)	0 (0)	
Dissatisfied	7 (9)	7 (12)	0 (0)	
Very dissatisfied	2 (3)	2 (3)	0 (0)	
The duration of assessment process in each shift (min)				.54
Mean (SD)	15.5 (10.2)	15.3 (9.0)	16.8 (14.6)	
Median (IQR)	15 (5-15)	15 (5-15)	10 (5-15)	
The duration of feedback in each shift (min) (orally or in text)				.13
Mean (SD)	19.0 (16.0)	17.7 (14.9)	24.6 (19.6)	
Median (IQR)	15 (5-15)	15 (2-28)	18 (8-28)	
Likelihood of forgetting to complete the assessments				.02
Mean (SD)	3.1 (1.3)	3.3 (1.3)	2.4 (1.1)	
Median (IQR)	3 (1-5)	3 (1-5)	3 (1-5)	
Present Method of the performance result, n (%)				—
Not seen	4 (5)	4 (7)	0 (0)	
With number (0‐100)	17 (23)	10 (17)	7 (50)	
With level (1-5)	53 (72)	44 (73)	9 (64)	
With number and visualized trend	31 (42)	19 (32)	12 (86)	
With number, visualized trend, and complete rate	36 (49)	24 (40)	12 (86)	
Can you identify whether the performance trend is improved or worsened from the assessment result?				<.001
Mean (SD)	3.1 (1.3)	2.7 (1.1)	4.7 (0.6)	
Median (IQR)	3 (1-5)	2 (1-3)	5 (5-5)	
Can you review and respond to the feedback within 24 hours?				<.001
Mean (SD)	2.7 (1.3)	2.2 (0.9)	4.5 (0.9)	
Median (IQR)	2 (1-3)	2 (1-3)	5 (4-5)	
Are you able to individualize the training program based on performance results?				<.001
Mean (SD)	2.7 (1.1)	2.3 (0.8)	4.4 (0.8)	
Median (IQR)	3 (2-4)	2 (1-3)	5 (4-5)	
Can you identify the required clinical scenarios and the assessments needed for each trainee?				<.001
Mean (SD)	2.6 (1.3)	2.1 (0.8)	4.8 (0.4)	
Median (IQR)	2 (1-3)	2 (2-2)	5 (5-5)	

a Not applicable.

b RRC: indicated residency review committee.

Further statistical analysis indicated that the use of MB platforms was significantly associated with a 2.08-point increase (95% CI 1.73‐2.43, *P*=.002) in the ability to individualize training programs based on performance results (Table 3).

**Table 3.** Regression analysis for individualization of training program.

Variables	OR^a^ (95% CI)	*P* value
Mobile-based	2.08 (1.73‐2.43)	.02
Number of trainees	<0.01 (–0.07 to 0.06)	.78
Number of trainers	<0.01 (–0.02 to 0.02)	.89

a OR: odds ratio

## Discussion

### Principal Findings

This pilot study found that mobile-based programs more frequently used visualized performance trends (85.71%) than paper- and web-based programs (73.33%), enhancing trainees’ understanding (mean 4.71, SD 0.61 vs mean 2.72, SD 1.06; *P*<.001). Mobile-based platforms also enabled faster review of ad hoc responses within 24 hours (mean 4.5, SD 0.85 vs mean 2.22, SD 0.88; *P*<.001) and better supported individualized training in alignment with CBME principles (mean 4.36, SD 0.84 vs mean 2.27, SD 0.78; *P*<.001). Statistical analysis indicated a significant association between MB assessments and improved training individualization (2.08-point increase, 95% CI 1.73‐2.43; *P*=.002), suggesting mobile-based platforms facilitate more timely and adaptive program adjustments than paper- and web-based (Table 3).

### Comparison With Previous Work

Since 2009, CBME has become a global standard in medical training [1]. Oudkerk Pool et al [14] highlight that competency judgments are formed through an iterative process of acquiring and synthesizing evidence, underscoring the need for structured, multisource assessments. However, effective CBME implementation requires more than data collection; it demands meaningful integration and interpretation among instructors, residents, and program coordinators, including RRC hosts and CCC members [14,16].

Despite advancements in assessment methods, paper- and web-based evaluations remain prevalent in Taiwan’s residency programs (Table 2). These static, retrospective tools limit real-time performance tracking and individualized feedback. Chan et al [16] demonstrated that programmatic workplace-based assessments, such as the McMaster Modular Assessment Program (McMAP), improve competency evaluations by replacing single-assessor recall with multisource, continuous feedback. Likewise, our findings suggest that mobile-based platforms offer a promising alternative by enabling timely communication, visualizing performance trends, and tracking clinical exposures in real time, aligning with CBME’s emphasis on progressive competency tracking.

However, while convenience is improved, adopting a mobile-based platform alone does not guarantee the effective integration of summative performance trends. As Oudkerk Pool et al [14] emphasize, competency judgments require active evidence synthesis rather than passive data aggregation. Therefore, mobile-based platforms must be supported by faculty training and institutional commitment to continuous assessment. Beyond technology, mobile-based platform development reflects institutional investment in assessment culture. Successful implementation requires standardized tools, integration, and a culture that embraces real-time feedback. Chan et al [16] highlight that workplace-based assessment systems like McMAP not only streamline logistics but also normalize structured, frequent evaluations, shaping residency training [12]. This may explain why, in our study, the mobile-based platform yielded higher satisfaction and a more tailored training experience compared with the paper- and web-based group.

Compared with mobile-based assessment evaluations, paper- and web-based assessments, though practical, are often less accessible, time-consuming, and associated with lower compliance [19]. Mobile-based platforms provide easy access and real-time feedback, allowing trainers and trainees to review performance trends anytime and anywhere. In addition, their integration with smartphone features—such as cameras, note apps, calendars, GPS, and barcode scanners—enhances efficiency and usability [18,19,21-23]. However, while mobile-based platforms improve accessibility, Walsh [5] noted that mobile learning in medical education may also introduce distractions, potentially affecting engagement and focus during training sessions.

Previous studies have demonstrated the feasibility and effectiveness of mobile-based assessment tools in medical training. Nethala et al [11] found that mobile apps enable real-time assessment and individualized skill training in urology residency programs, facilitating structured competency tracking. Similarly, Sung and Park [22] reported that a mobile-based training program improved nurses’ competence through enhanced accessibility and interactive learning. Green highlighted the cost-effectiveness and usability of smartphone platforms for surgical resident evaluations, reinforcing the practical benefits of mobile-based assessments in medical education [23].

In our study, while assessment and feedback durations did not differ significantly between mobile-based and paper- and web-based platforms, mobile-based assessments yielded higher satisfaction and a lower frequency of missed evaluations (*P*<.001 and *P*=.02, respectively; Table 1). These findings suggest that the convenience and accessibility of mobile platforms may enhance trainer engagement and encourage more consistent assessment documentation.

Beyond technological advantages, integrating multisource evaluations into visualized trend tracking on mobile-based platforms may enhance participant engagement, foster active participation, and support continuous improvement. According to self-determination theory, providing trainees with tools for real-time performance monitoring fosters autonomy, competence, and relatedness, key psychological needs that enhance motivation and self-directed learning [13]. When trainees and trainers can instantly track progression and performance trends, they gain a clearer perception of their self-determination (Figure 2), which may drive greater accountability and active participation in their own professional growth. This aligns with Association for Medical Education in Europe Guide No. 59, which highlights the role of real-time feedback in enhancing intrinsic motivation and engagement in competency development [12,13].

Our survey found that although some web-based programs included visualized trends and completion rates, mobile-based programs had a significantly greater impact on both trainers and trainees (mean 2.3, SD 0.8 vs mean 4.4, SD 0.8; *P*<.001; Table 2). This advantage likely stems from the mobile-based platform’s accessibility, enabling real-time review compared with paper- and web-based assessments, which are typically evaluated only during biannual or quarterly CCC meetings [24]. Delayed review in paper- and web-based evaluations may hinder timely performance adjustments, whereas immediate access to evaluation results in mobile-based programs enables trainees to modify their performance on the next shift, reinforcing self-directed learning [13].

The observed advantages of mobile-based platforms should not be attributed solely to their technology but rather to the broader institutional commitment to structured assessment and a culture of continuous feedback. While mobile-based platforms facilitate real-time performance tracking and self-directed learning, their effectiveness depends on institutional investment in faculty training, standardized assessment frameworks, and integration within CBME structures [4,16]. The shift from paper- and web-based biannual review model [24] to continuous monitoring reflects not just technological convenience but a deeper transformation in how competency is assessed and supported. As previous studies highlight, frequent, structured feedback is essential for meaningful competency development [12,15], and the adoption of MB platforms likely signifies an institutional prioritization of learner-centered training and assessment rigor rather than merely a technological upgrade. This shift, reinforced by evidence-based coaching and real-time decision-making, aligns with a growing recognition that assessment culture, not just digital tools, drives improved training experience [13,16].

The higher satisfaction with mobile-based platforms compared with paper- and web-based (*P*<.001, Table 2) may be attributed to their ability to provide real-time accessibility, structured assessments, and visualized performance trends, benefiting trainees, trainers, and RRC hosts. For trainees, mobile-based platforms enabled immediate feedback and continuous performance tracking, allowing timely adjustments rather than relying on periodic CCC reviews. This aligns with CBME principles by supporting self-directed learning and competency progression. In addition, mobile-based platforms facilitated easier identification of required clinical scenarios and assessments (*P*<.001), which may contribute to a more structured training experience.

For trainers, mobile-based platforms allowed more flexible and timely assessments, reducing recall bias and documentation burden. The ability to complete evaluations outside clinical hours (*P*=.02; Table 2) suggests increased convenience and consistency in providing feedback. The integration of smartphone tools, such as cameras and note apps, may have contributed to streamlined documentation and more structured evaluations [21]. For RRC hosts, mobile-based platforms provided a longitudinal perspective on trainee progress, reducing reliance on episodic assessments and allowing competency decisions to be based on continuous performance data rather than retrospective impressions [14,16]. The findings suggest that mobile-based platforms improve accessibility and assessment efficiency, reflecting a shift toward more continuous, data-driven evaluation in residency training.

The preference for mobile-based platforms in tailored training (*P*<.001; Table 2) stems from their ability to provide real-time feedback, trend visualization, and individualized learning, aligning with CBME’s emphasis on continuous assessment [2,4]. Unlike paper- and web-based periodic evaluations, mobile-based platforms empower trainees with self-directed learning and immediate performance adjustments while enabling trainers to deliver data-driven coaching with minimized bias [12,16]. For RRC hosts, mobile-based platforms enhance longitudinal competency tracking, overcoming the limitations of CCC’s fixed evaluation intervals and allowing timely interventions [1,3,24]. However, their success depends on structured implementation, faculty engagement, and integration within CBME frameworks to ensure meaningful assessment without cognitive overload [5,6,17].

Overall, while mobile-based platforms show promise in improving feedback timeliness and training individualization in our pilot study, further research is needed to evaluate their long-term impact on competency development, clinical outcomes, and residency training culture.

### Highlights

First, mobile-based platforms provide real-time feedback and continuous performance tracking, complementing scheduled CCC reviews and addressing paper- and web-based limitations in integrating multisource evaluation results. Second, structured mobile-based evaluations improve engagement and training individualization, offering more timely and adaptive learning opportunities that better align with CBME principles compared with paper- and web-based platforms. Finally, successful integration depends on faculty training and strategic implementation, enhancing assessment validity, competency tracking, and institutional commitment to resident development, ultimately leading to greater satisfaction.

Structured mobile-based evaluations improve engagement and training individualization, offering more timely and adaptive learning opportunities that better align with CBME principles compared with paper- and web-based platforms. Successful mobile-based integration depends on faculty training and strategic implementation, enhancing assessment validity, competency tracking, and institutional commitment to resident development, ultimately leading to greater satisfaction.

### Limitations

First, the study results were based on a convenience sample from an internet survey. However, enrollment only covered 62.22% of the emergency residency training sites in the country. Second, the survey results indicated that only 14 out of 74 responses applied mobile-based assessment. Further investigation to evaluate mobile-based assessment will be needed as more training programs start being delivered through the mobile interface. Finally, in the survey, participants provided responses based on their experience with the interface’s ability to facilitate tailored training adjustments, rather than on direct evidence of its actual implementation. Further research is needed to assess the long-term impact of mobile-based platform implementation on training outcomes.

The survey results indicated that only 14 out of 74 responses applied mobile-based assessment. Further investigation to evaluate mobile-based assessment will be needed as more training programs start being delivered through the mobile interface.

In the survey, participants provided responses based on their experience with the interface’s ability to facilitate tailored training adjustments, rather than on direct evidence of its actual implementation. Further research is needed to assess the long-term impact of mobile-based platform implementation on training outcomes.

### Conclusion

In conclusion, the mobile-based interface emerges as a dynamic and effective platform for emergency residency training programs. It facilitates rapid updates and individualized program modifications, thereby increasing the engagement and satisfaction of trainers, trainees, and other stakeholders. Contrarily, the conventional paper- and web-based methods face limitations due to prolonged review periods in committee meetings, which may lead to delays in crucial program modifications and impede the progression of the program. The adoption of mobile-based technology in this context demonstrates its capacity to greatly enhance the efficiency and efficacy of CBM, particularly in making tailored adjustments. This technology also promotes better-informed and more collaborative interactions among all involved parties.

## Supplementary material

10.2196/57216 Multimedia Appendix 1 Internet survey form.

## Abbreviations

CBME: competency-based medical education
CCC: Clinical Competency Committee
McMAP: McMaster Modular Assessment Program
RRC: Residency Review Committee
