# Supplementary material for: Evaluating Tailored Learning Experiences in Emergency Residency Training Through a Comparative Analysis of Mobile-Based Programs Versus Paper- and Web-Based Approaches: Feasibility Cross-Sectional Questionnaire Study
Source: JMIR Med Educ. 2025 Jul 24;11:e57216. doi: 10.2196/57216 (PMC12288858; doi:10.2196/57216)
Supplement: Multimedia Appendix 1 [file mededu-v11-e57216-s001.docx]

Internet survey questions for emergency training programs.

| What is the level of your training site?   1. District hospital 2. Regional hospital 3. Medical center |  |  |  |
| --- | --- | --- | --- |
| What is your role in the training program?   1. Junior resident (R1/R2) 2. Senior resident (R3/R4) 3. RRC host or CCC members 4. Director of the department 5. Faculties |  |  |  |
| Assessment interface: | Paper-based | web-based | mobile-based |
| Are you satisfied with the assessment interface? |  |  |  |
| How long do you assess the resident for during each shift? (including the recording period) |  |  |  |
| How long does it take you to give feedback to your resident during each shift? |  |  |  |
| How often do you forget to complete the assessment during each shift? |  |  |  |
| The present method of performance result:   1. Not seen 2. With numbers 3. With level 4. With numbers and visualized trend 5. With numbers, visualized trend, and completion rate |  |  |  |
| Can you identify whether the performance trend is improved or worsened from the assessment result? |  |  |  |
| Can you review and respond to the feedback within 24 hours? |  |  |  |
| Are you able to individualize the training program based on performance results? |  |  |  |
| Can you identify the required clinical scenarios and the assessments needed for each trainee? |  |  |  |
